# A Population-Based Study of U.S. Trends in Selected Congenital Anomalies (2016–2023) and Socio-Demographic Disparities: A CDC WONDER Analysis

**DOI:** 10.3390/children13020192

**Published:** 2026-01-29

**Authors:** Mahmoud Ali, Ramesh Vidavalur, Naveed Hussain

**Affiliations:** 1 West Virginia University Children Hospital, Morgantown, WV 26505, USA; 2 Cayuga Medical Center of Ithaca, Ithaca, NY 14850, USA; 3 Connecticut Children’s Medical Center, Farmington, CT 06032, USA

**Keywords:** congenital anomalies, birth defects, CDC WONDER, socio-demographic disparities, maternal risk factors, prevalence, United States, joinpoint regression

## Abstract

**Highlights:**

**What are the main findings?**

- Overall prevalence of selected congenital anomalies was 3.3 per 1000 live births in 2023.
- Higher risk was observed with pre-pregnancy diabetes, advanced maternal age, tobacco use, and higher BMI and among American Indian/Alaska Native infants.

**What are the implications of the main findings?**

- Targeted preconception and early prenatal interventions may reduce modifiable risks and address disparities.
- Continued national surveillance can inform policy and resource allocation for prevention and care.

**Abstract:**

Background: Congenital anomalies are influenced by genetic and environmental factors. While interventions including folic acid supplementation have reduced neural tube defects, data on modifiable socio-demographic risk factors remain limited. Aim: This study aimed to assess variation in the prevalence of selected congenital anomalies across the United States according to socio-demographic factors. Methods: A population-based analysis was conducted using CDC-WONDER natality data from 2016 to 2023. Included anomalies were anencephaly, spina bifida, cyanotic heart disease, diaphragmatic hernia, omphalocele, gastroschisis, limb reduction, cleft lip/palate, Down syndrome, chromosomal disorders, and hypospadias. Associations with maternal age, BMI, race, tobacco use, diabetes, and fertility treatments were analyzed. Prevalence rates were calculated per 1000 live births. Relative risks (RRs) and 95% confidence intervals (CIs) were estimated. Joinpoint regression was used to assess annual percent changes (APCs), with *p* < 0.05 considered significant. Results: Among 3,482,944 singleton live births in 2023, the overall prevalence of the selected congenital anomalies was 3.3 per 1000. Compared to Caucasian mothers, risk was lower in Asian (RR 0.57; 95% CI: 0.52–0.63) and Black (RR 0.81; 95% CI: 0.76–0.85) infants and higher in American Indian/Alaska Native infants. Significant risk factors included pre-pregnancy diabetes (RR 2.41; 95% CI: 2.16–2.69), maternal age > 45 (RR 2.95; 95% CI: 2.36–3.69), and tobacco use (RR 1.78; 95% CI: 1.64–1.94). A significant decline in prevalence was observed from 2016 to 2023 (APC: −0.6%; 95% CI: −1.1 to −0.2; *p* = 0.006). Conclusions: Significant disparities and modifiable maternal risk factors were associated with the prevalence of selected congenital anomalies in the U.S. from 2016 to 2023. A modest statistically significant decline in overall prevalence was observed during the study period, supporting the importance of continued national surveillance and targeted preconception and prenatal interventions to reduce risk and address inequities.

## 1. Introduction

Congenital anomalies are structural or functional abnormalities that arise in utero [1]. These conditions may be identified in utero, at birth, or later in life if they are initially overlooked [1]. According to the European Surveillance of Congenital Anomalies (EUROCAT), the prevalence of congenital anomalies was estimated at 23.9 per 1000 births between 2003 and 2007 [2]. The associated perinatal mortality is approximately 9.2 per 10,000 births [3]. Globally, congenital anomalies account for 4.4% of neonatal deaths, according to the Global Burden of Disease (GBD) report [4], and they rank as the fifth leading cause of death in children under five years of age [5].

In the United States, congenital anomalies account for approximately 15–30% of pediatric hospitalizations [3]. Proposed etiologies include genetic predisposition, environmental exposures such as chemicals or infectious agents, and maternal socioeconomic or demographic factors. Often, these influences interact, though the precise causes remain largely unknown [3,4].

In this study, we examined the heterogeneity in the prevalence of congenital anomalies across socio-demographic groups, aiming to inform prevention strategies, evaluate the effects of reproductive health legislation, and describe the burden of modifiable risk factors.

## 2. Materials and Methods

This cross-sectional population-based study utilized aggregated birth certificate data from the publicly available CDC-WONDER natality database, focusing on selected congenital anomalies reported between 2016 and 2023. The database includes statistics on births that occurred within the United States, based on officially issued birth certificates [6]. Data are compiled at the county level through the Vital Statistics Cooperative Program (VSCP) and maintained by the National Center for Health Statistics (NCHS). All live births reported across 57 vital statistics jurisdictions were included. Maternal characteristics in this dataset are categorized into six racial groups: American Indian or Alaska Native (AIAN), Asian, Black or African American, Native Hawaiian or Other Pacific Islander (NHOPI), White, and more than one race. This subset represents congenital anomalies available within the CDC WONDER natality dataset and is not intended to capture the full spectrum of congenital malformations.

The reported anomalies included anencephaly, meningomyelocele/spina bifida, cyanotic congenital heart disease, congenital diaphragmatic hernia, omphalocele, gastroschisis, limb reduction defects, cleft lip with or without cleft palate, cleft palate alone, Down syndrome, suspected chromosomal disorders, and hypospadias. Maternal factors such as age, body mass index (BMI), race, and other sociodemographic variables were analyzed for potential associations. Prevalence rates were pooled and reported per 1000 live births. Bivariate analyses were performed to evaluate the influence of race, maternal BMI, tobacco use, maternal age, and use of assisted reproductive technologies. Relative risks (RR) with 95% confidence intervals were calculated using MedCalc Statistical Software, version 19.2.6.

Joinpoint regression analysis (version 5.3.0, National Cancer Institute, Rockville, MD, USA. 2024) was used to estimate annual percent changes (APC), with statistical significance set at *p* < 0.05. This method, a form of piecewise linear regression, models time series data by fitting consecutive linear segments—each with its own slope—to detect significant trend shifts over time. The analysis was limited to a maximum of nine joinpoints to identify the fewest statistically significant changes in trend.

For each segment, the APC was calculated as the slope of the regression line, expressed as a percentage, to quantify annual changes in the prevalence of selected congenital anomalies. Corresponding 95% confidence intervals (CI) were computed to assess the magnitude and precision of these trends. In the model, congenital anomaly prevalence rates served as the dependent variable, with year of birth as the independent variable.

## 3. Results

In 2023, among 3,482,971 reported singleton live births, the overall prevalence of selected congenital anomalies was 3.3 per 1000 live births.

**Figure 1:**
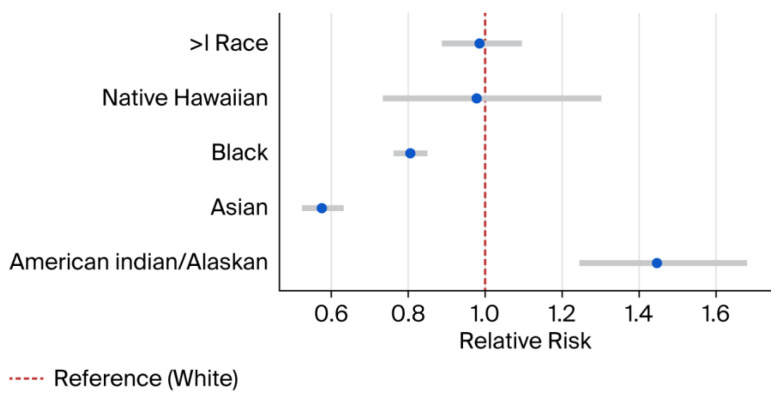
Relative risk estimates by race. Blue dots represent RR and error bars represent 95% CI. RR = Relative Risk. CI = Confidence interval. > = More than.

Compared to White infants, the risk was significantly lower among Asian infants (RR 0.57; 95% CI: 0.52–0.63) and Black infants (RR 0.81; 95% CI: 0.76–0.85) (Figure 1). In contrast, American Indian/Alaska Native infants had a higher prevalence (RR 1.45; 95% CI: 1.24–1.68).

**Figure 2:**
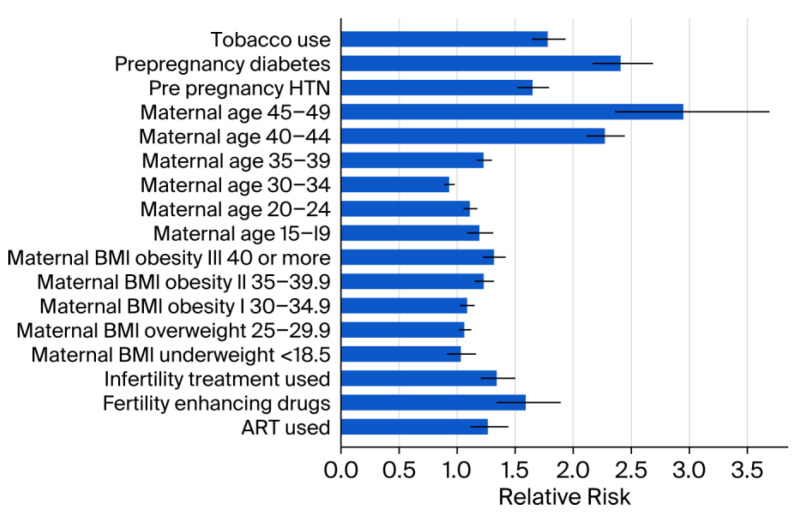
Relative risk estimates by other socio-demographic factors. Error bars represent 95% CI. ART = Assisted reproductive technology. BMI = Body mass index. CI = Confidence interval. HTN = Hypertension. < = less than.

Medical and demographic factors were associated with increased prevalence, including pre-pregnancy diabetes (RR 2.41; 95% CI: 2.16–2.69; *p* < 0.001) and advanced maternal age > 45 years (RR 2.95; 95% CI: 2.36–3.69; *p* < 0.001). In addition, the lifestyle factor tobacco use was associated with increased prevalence (RR 1.78; 95% CI: 1.64–1.94; *p* < 0.001) (Figure 2).

**Figure 3:**
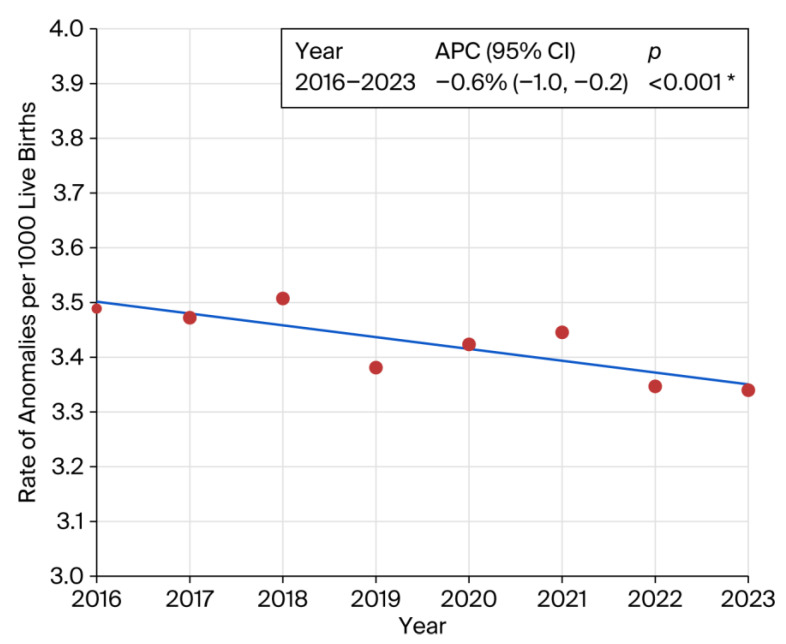
Joinpoint regression showing prevalence trend of congenital anomalies. Inset represents annual percent change (APC). * indicates that the Annual Percent Change (APC) is significantly different from zero at the alpha = 0.05 level. Final Selected Model: 0 Joinpoints.

Stratification by BMI revealed a significant increase in congenital anomalies among individuals in the overweight and obese categories compared to the reference BMI range of 18.6–24.9 (Supplementary Table S1). Similar elevated risks were observed for pre-pregnancy hypertension (RR 1.65; 95% CI: 1.52–1.79; *p* < 0.001) and the use of fertility-enhancing drugs (RR 1.59; 95% CI: 1.34–1.89; *p* < 0.001). Pre-pregnancy diabetes was associated with a substantially increased risk of congenital anomalies (RR 2.41; 95% CI: 2.16–2.69). A statistically significant downward trend in overall prevalence was observed from 2016 to 2023 (APC: −0.6%; 95% CI: −1.0 to −0.2; *p* < 0.001) (Figure 3).

## 4. Discussion

Congenital anomalies remain a global health priority due to their significant contribution to neonatal and childhood morbidity and mortality [3,4,5]. While high-income countries have reduced mortality related to congenital anomalies through advances in healthcare systems and better antenatal care, low- and middle-income countries (LMICs) continue to face high prevalence rates and poor survival outcomes [1,2,3,4,5]. In this study, we observed the following: (i) an overall prevalence of selective congenital abnormalities at 1 in 300 live births; (ii) a higher risk of congenital abnormalities in American India/Alaska Native infants and lower risk in Black and Asian infants; and (iii) a strong association between birth defects and several socio-demographic and lifestyle factors. Observed associations with maternal diabetes, obesity, and tobacco exposure are biologically plausible through pathways involving altered embryogenesis, placental dysfunction, oxidative stress, and impaired fetal growth and development.

Prior studies of associations between race and congenital anomalies have largely been restricted to individual states or specific anomaly types [7,8]. A large retrospective analysis conducted by Mohamed and Aly. highlighted place of birth as a contributing factor alongside sex and race in the occurrence of neural tube defects, abdominal wall defects, and congenital diaphragmatic hernia [9]. Our study represents the most comprehensive, large-scale, and up-to-date investigation to date examining the association between race and the prevalence of this full set of CDC-reportable congenital anomalies. Racial/ethnic differences in congenital anomaly prevalence may reflect a combination of social and structural determinants of health, including variation in access to preconception and prenatal care, maternal comorbidities, environmental exposures, and healthcare quality [7,8]. The elevated risk observed among AI/AN pregnancies may be influenced by these factors rather than purely biological causes [7,8,9]. Conversely, the lower observed prevalence among Asian and Black populations may reflect differences in baseline risk profiles and/or variation in screening, detection, and reporting [7,8,9]. These findings should be interpreted cautiously given known underreporting of congenital anomalies in birth certificate data.

One of the key socio-demographic risk factors identified in our study is the strong association between advanced maternal age (35–49 years) and an increased risk of congenital anomalies. Maternal age ≥ 45 years was associated with the highest risk of selected congenital anomalies among all maternal age groups. Our findings differ from earlier European studies that analyzed the link between advanced maternal age (AMA) and congenital anomalies [10,11,12], reporting a reduced risk in this age group [13]. In contrast, our results align with several previous reports affirming this association [14,15,16,17], underscoring the need for targeted prenatal care and genetic counseling for older expectant mothers. The elevated risk observed in this age group may be attributable to several factors, including a higher prevalence of comorbidities, increased environmental exposures, and more frequent use of assisted reproductive technologies [18,19,20,21,22]. Notably, the literature on AMA and congenital anomalies is inconsistent, with reports in both directions. Differences in anomaly definitions, surveillance methods, and adjustment for confounders (e.g., comorbidities and ART) may explain these discrepancies.

Similarly, maternal obesity emerged as another significant risk factor. Smaller-scale studies and a 2009 meta-analysis have reported a higher prevalence of congenital anomalies in individuals with elevated BMI [23,24,25]. Our findings reaffirmed this observation, showing that the prevalence of congenital anomalies increased consistently with higher maternal BMI. Potential mechanisms include metabolic disturbances, such as insulin resistance and hyperglycemia common in diabetes, as well as nutritional deficiencies; lower folate levels in obese pregnant women may reduce the protective effect of folic acid against neural tube defects [23].

Pre-pregnancy diabetes is a well-established risk factor for congenital anomalies. Experimental studies suggest that hyperglycemia during early embryogenesis can alter gene expression in critical cellular pathways [26,27,28,29,30], while the oxidative stress associated with a hyperglycemic intrauterine environment further elevates the risk of anomalies in the developing fetus [31,32]. Epidemiological evidence has consistently linked pre-pregnancy diabetes to a range of congenital anomalies, including congenital heart defects, oral clefts, and abnormalities affecting the central nervous, digestive, genitourinary, and musculoskeletal systems [33,34,35,36,37,38,39]. Unfortunately, the global prevalence of diabetes among women of reproductive age is on the rise [31,32]. A population-based study from Canada, conducted by Liu et al., reported that the prevalence of pre-pregnancy diabetes in women over 35 years nearly doubled—from 6.4 per 1000 live births in 2002 to 12.3 per 1000 in 2012. Over the same period, the proportion of congenital anomalies attributed to maternal pre-pregnancy diabetes also increased [37]. Findings from this study align with these patterns, showing more than a twofold increase in the risk of congenital anomalies among pregnancies affected by pre-pregnancy diabetes, underscoring the escalating scope and clinical significance of this public health issue. Preconception counseling for women with diabetes is essential, with a focus on achieving optimal glycemic control, ensuring adequate nutrition, and supplementing with folic acid [40]. In this high-risk population, early diagnosis of congenital anomalies—especially lethal forms—followed by timely pregnancy termination when appropriate, plays a critical role in mitigating adverse sequelae [41]. While the literature on pre-pregnancy hypertension and congenital anomalies is limited [42,43], our study identified a significantly increased risk among women with a pre-existing diagnosis of hypertension (RR 1.65; 95% CI: 1.52–1.79; *p* < 0.001).

In high-income countries, smoking prevalence among pregnant women is estimated at around 10% [44], with some reports ranging from 6% to 22% [45]. In our report, 3% of singleton live births had documented maternal tobacco use, while 0.37% were recorded as unknown Our findings are consistent with a recent systematic review and meta-analysis conducted by Sabbagh et al., which reported a twofold increase in the risk of congenital anomalies among infants born to mothers who smoked during pregnancy [46]. In our cohort, smoking was associated with a 78% elevated risk, underscoring the need for targeted interventions to reduce prenatal tobacco exposure during pregnancy.

Infertility treatments have been identified as contributing to an increased risk of congenital anomalies [47]. In our study, women who used fertility-enhancing drugs had a significantly higher risk of having infants with congenital anomalies compared to those who did not. In this study, the observed risk surpasses earlier estimates, which suggested only a 1% to 2% increase in congenital anomaly prevalence following infertility treatment [47,48].

We identified a statistically significant downward trend in the prevalence of congenital anomalies from 2016 to 2023 (APC: −0.6%; 95% CI: −1.1 to −0.2; *p* 0.006). This finding aligns with a Canadian population-based report, which documented a decline in prevalence from 50.7 per 1000 live births in 2002–2003 to 41.5 per 1000 in 2012–2013 [37]. Another European study reported statistically significant increase in twelve congenital anomaly subgroups, mainly congenital heart defects and decrease in limb reduction defects the 2003–2012 period [49]. It is important to remember that the downward trend in APC noted in our study encompassed all birth-certificate-reported congenital anomalies as one entity, while, within each anomaly category, there may have been variations, with some increases and some decreases [49].

The strengths of this study include the use of recent large-scale population-based national data, the inclusion of sociodemographic factors in the analysis, and the accurate description of trends. By examining key sociodemographic factors in depth, the study provides actionable insights that can inform current healthcare policies and practices. The findings are particularly valuable for policymakers and healthcare providers aiming to strengthen prevention strategies and enhance prenatal care—both essential for designing targeted interventions and improving public health outcomes.

However, this study has several limitations. The dataset is based on birth certificate records, which may underreport or misclassify congenital anomalies, and it includes only a limited range of conditions, potentially omitting less common or later-diagnosed anomalies, thereby restricting the scope of analysis. It has been shown that, while birth certificates are relatively accurate for structural defects such as gastroschisis that are apparent at time of birth, they significantly underreport conditions that manifest later, such as heart defects and chromosomal abnormalities, which are captured in active surveillance registries. which may underreport. The analysis also focuses solely on prevalence and does not account for long-term survival outcomes or regional differences in environmental exposures, healthcare access, or care quality across the United States. Because this analysis relied on aggregate CDC WONDER natality data, we were unable to perform fully adjusted multivariable modeling; therefore, associations observed for maternal risk factors, including hypertension, may reflect residual confounding by correlated maternal characteristics (e.g., BMI) and/or medication exposures. In addition, the use of aggregated data precluded conducting internal validation studies or sensitivity analyses to quantify the degree of underreporting. Although this study includes all 57 vital statistics jurisdictions, we did not perform jurisdiction- or region-specific analyses, and state-level differences in prenatal care access and reproductive health policies during the study period may have influenced screening, diagnosis, and ascertainment of congenital anomalies. Despite these limitations, this up-to-date analysis offers critical insights into the sociodemographic patterns underlying congenital anomaly prevalence. It highlights the importance of targeted prevention strategies to improve maternal health and enhance prenatal care among populations at higher risk.

## 5. Conclusions

The findings of this study underscore the need for a periconceptional multidisciplinary approach to mitigate the risk of congenital anomalies, including preconception and early prenatal counseling, targeted surveillance for higher-risk pregnancies (e.g., pre-pregnancy diabetes and maternal age > 45 years), and interventions addressing modifiable lifestyle factors such as tobacco use and elevated BMI. Racial disparities were also observed, including higher prevalence among American Indian/Alaska Native infants in this dataset, underscoring the need for equitable, culturally responsive prevention strategies and access to high-quality preconception and prenatal care. Race should not be interpreted as a biologic causal risk factor; rather, these differences likely reflect social and structural determinants of health and inequities in access to care. Further research is warranted to examine regional variation and potential residual confounding. Ultimately, these findings contribute to a broader understanding of congenital anomalies and support more effective, data-driven public health initiatives.

## Data Availability

This cross-sectional population-based study utilized aggregated birth certificate data from the publicly available CDC-WONDER database.

## Supplementary Materials

The following supporting information can be downloaded at: https://www.mdpi.com/article/10.3390/children13020192/s1.

## Abbreviations

The following abbreviations are used in this manuscript:

APC	annual percent change
ART	assisted reproductive technology
BMI	body mass index
CI	confidence interval
NCHS	National Center for Health Statistics
RR	relative risk
VSCP	Vital Statistics Cooperative Program
